# Genetic knowledge and attitudes towards genetic testing among final-year medical students at a public university in Ecuador

**DOI:** 10.3389/fmed.2024.1363552

**Published:** 2024-06-19

**Authors:** Damary S. Jaramillo-Aguilar, Katherine Simbaña-Rivera

**Affiliations:** ^1^Escuela de Medicina, Facultad de Ciencias Médicas, Universidad de Cuenca, Cuenca, Ecuador; ^2^Centro de Investigación para la Salud en América Latina (CISeAL), Facultad de Medicina, Pontificia Universidad Católica del Ecuador (PUCE), Quito, Ecuador

**Keywords:** genetics, knowledge, attitude, medical students, Ecuador

## Abstract

**Background:**

Genetics plays a crucial role in the field of medicine, offering numerous applications. However, health professionals often have insufficient knowledge in this area. Therefore, it is essential to provide appropriate genetics education during university studies.

**Aim:**

This study aimed to assess the knowledge and attitudes towards genetic testing among final-year medical students at a public university in Ecuador.

**Methods:**

A cross-sectional study was conducted involving final-year medical students from a public university in Ecuador. The third version of the Genetic Literacy and Attitudes Survey was administered between April and May 2022. The study examined sociodemographic characteristics, genetic knowledge, and attitudes towards genetic testing.

**Results:**

The study included 153 medical students, of which 58.2% identified as female. Most participants fell within the age range of 22 to 25 years old (85.0%). Regarding genetic knowledge, three-quarters of the participants (75.2%) demonstrated intermediate proficiency, while only 9.80% possessed a high level of knowledge. Attitudes towards the clinical and therapeutic applications of genetics, scientific advancements, access to conventional medicine, and other related topics were found to be appropriate.

**Conclusion:**

The findings suggest that most final-year medical students at a public university in Ecuador have intermediate genetic knowledge and hold appropriate attitudes towards genetic testing. However, higher education institutions should conduct a comprehensive analysis and restructure their curricula to better prepare students for the medical and technological challenges of the 21st century.

## 1 Introduction

Genetics is a discipline within biology that studies the aspects of gene structure and function, genetic variability and inheritance in living organisms, at the cellular, molecular, and population levels (1). In the 1980s, the Human Genome Project (HGP) was launched with the objective of deciphering the chemical composition of the human genetic code in order to identify genes associated with both normal biology and common and rare diseases (2). This project revolutionized biological sciences and emphasized the fundamental role of genetics in life and the future of health sciences. Since then, technology and research in genetics have advanced significantly. Although genetics has allowed to understand the impact of preventing disease and improving the health of population on health systems, it continues growing at an unprecedented pace. As a result of this, genetics has vast applicability in medicine, serving as a diagnostic, preventive, therapeutic, and adequate surveillance tool for patients, their families, and the population (3).

Nevertheless, several studies have shown that health professionals have poor knowledge of genetics, personalized medicine, and its advances, as well as genetic tests and their interpretations, and genetic counselling and its indications (4). Such knowledge directly affects practice, resulting in patients not being referred to specialized services and receiving inadequate treatment (5, 6). In this regard, undergraduate training in genetics is crucial. Nonetheless, the crux of the matter lies in that the teaching-learning process in genetics has numerous shortcomings (7, 8). In Latin America, the exploration of genetics education within undergraduate medical programs and its investigation among the general population remains limited. This scarcity can be attributed to various factors including health disparities experienced by the population, a paucity of genetic resources in both public and private domains, the absence of regulations governing the handling of genetic information, and insufficient engagement of healthcare professionals in the subject (9). Following an extensive literature review, three studies concerning genetics and genetic testing among undergraduate medical and non-medical students were conducted in Argentina, Mexico, and Ecuador, respectively (10–12). Notably, Brazil and Mexico have demonstrated significant efforts in exploring this subject among healthcare professionals compared to other countries in the region in recent years (13–16).

One study, as previously mentioned, was undertaken in Ecuador, aiming to evaluate the fundamental knowledge of genetics and its implications in specific diseases among undergraduate students not majoring in biology or medicine (12). The assessment tool employed was validated for non-specialized populations and comprised 18 items with binary options (true or false). The students attained a final score below 70 points, ranging from 45 to 87, which the authors deemed satisfactory. However, despite the diverse participation of students from both public (3) and private (4) Ecuadorian Higher Education Institutions (HEIs) in Quito, these findings are not representative or generalizable to other HEIs, letting alone specialized populations, such as graduate or undergraduate medical students. Given the variability in academic curricula, these groups tend to possess a deeper understanding of the subject matter. Additionally, socioeconomic factors and practical experience could contribute to this disparity. Nevertheless, these results establish a foundational insight into genetic knowledge within the country, highlighting the necessity for improving genetic literacy across the general population (12). Moreover, the insufficient emphasis placed on this discipline within the health sector is noteworthy, particularly considering that in the majority of Ecuadorian HEIs, this course is included in the curriculum (17).

Ecuadorian medical education system consists of public and private HEIs. Undergraduate and postgraduate programs are offered by the majority of them. Students are admitted on HEIs if they pass admission exams. In case of undergraduate programs, students must take a national and the university of interest’s health sciences exams. Curricula varies over public and private HEIs, but all of Ecuadorian medical schools share objectives and competencies in order to students develop medical, clinical and surgical knowledge, attitudes and skills. Medical education programs’ graduation requirements include to complete 5 years between educational and professional training and 12 months of clinical training inside a hospital (17). Postgraduate programs are available in Ecuador, but they are not aim of discussion of this work.

Genetics is part of the medical students’ educational training and it is covered during the first 3 years of the program as well as other HEIs around the world (18, 19). Contents are based on cellular transcription, replication and translation, cell differentiation, Mendel’s experiments, monogenic, polygenic and sex chromosomes inheritance, multiple allelism, lethal genes, hybridism and polyhybridism. These last two topics are not applied to human health, but they are taught as part of theoretical knowledge. Although these contents are necessary to understand the basis of the subject, diagnostic and therapeutic applicability of genetics is not studied in detail. Only certain techniques or procedures are mentioned as such as microscopy, live-cell imaging, cellular and molecular fractionation and gene expression and function studies.

Therefore, the main objective of this research study was to determine the knowledge and attitudes towards genetic testing among final-year medical students at a public University of Ecuador.

## 2 Materials and methods

### 2.1 Study design and participants

A cross-sectional study was conducted using an online survey applied through Google Forms platform, which was available from April 15 to May 7, 2022.

Participants were students currently enrolled in their final year of medical school of a public university in southern Ecuador. According to the official register of the University, the whole population of final-year medical students was 252.

### 2.2 Sample size determination and sampling technique

Based on the population and assuming a response rate of 50%, a 95% confidence level and a 5% margin error, a sample size of 153 respondents was calculated. To obtain a representative sample of the population while considering the time constraints and limited engagement of students in research activities, this sample size was chosen.

A simple random sampling technique was employed to select participants, utilizing the lottery method. Upon distribution of the questionnaire, students were given a period of 7 days to respond. In cases where students did not respond within this timeframe, the questionnaire was redistributed to new participants using the same sampling method. Furthermore, non-responding students were excluded from subsequent samplings.

This process was repeated three times until the desired sample size was achieved. The survey was initially sent to 153 students, resulting in an initial response rate of 80.39%. Subsequently, an additional 30 individuals were contacted, with a response rate of 83.33%. At the end, the questionnaire was distributed to 5 more individuals, yielding a response rate of 100%. In total, the survey was sent out to 188 individuals to reach the sample size required.

Throughout this period, responses were monitored, and email reminders were sent every 3 days to encourage participation of the selected students.

### 2.3 Inclusion and exclusion criteria

The study included students who voluntarily and willingly agreed to participate, who answered the questionnaire within the deadline. Foreign students who were conducting clinical, virtual, or in-person exchanges at the institution were excluded.

### 2.4 Questionnaire and data measurement

The third version in Spanish of the Genetic Knowledge and Attitude Survey (iGLAS 3) was used. This is a structured instrument that was qualitatively validated by Chapman et al. and was presented at The International Conference on Psychology and Education (20). The iGLAS 3 aimed to assess knowledge of genetics and attitudes towards genetic testing in general and specific populations. It underwent three stages of validation in a sample of 5,404 individuals, ranging from 18 years and above, from 78 countries, including academics, professionals, university students and general population (21).

The structure of iGLAS 3 consists of three general sections: (1) sociodemographic and additional data, with a total of 20 items; (2) genetic knowledge, with a total of 11 heritability estimate items and 20 theoretical questions; and (3) attitudes towards genetic testing, with 41 items for opinions, 6 vignettes and 2 neuromyths (21). However, not all the items were utilized in this study due to differences between the target population. For the purposes of this study, the sections on sociodemographic information and attitudes consisted of 6 and 10 items, respectively. Also, the questions regarding the genetic knowledge self-assessment were added to the knowledge section.

The survey was sent out via the institutional emails of the selected final-year medical students and it was administered by the students themselves, with the information provided by the Dean’s Office of the Faculty of Medical Sciences at the public University. To ensure that participants completed the entire questionnaire, all the items were mandatory to fill out. If a question was missing, the participant was unable to continue to the next section.

The sociodemographic characteristics of the population were analyzed, as well as their knowledge and attitudes towards genetic testing.

#### 2.4.1 Sociodemographic characteristics

Regarding sociodemographic characteristics, six variables were considered. Gender was divided into female, male, non-binary, and “prefer not to say,” while age was grouped into three subgroups: 22–25 years old, 26–30 years old, and over 30 years old. The number of children was divided into four categories: 0, 1, 2, and more than 3. Participants with children were also asked about them and their actual age. Regarding religion, participants were allowed to decide whether they wanted to answer or not, and several options were offered, including agnostic, atheist, christian, other religions, and no religion.

#### 2.4.2 Knowledge towards genetics

Genetic knowledge was explored into three linked parts. First, participants were asked about their level of genetic knowledge through a self-assessment on a scale of 0 to 100 which was classified in the same way as the next part.

Second, knowledge about genetics properly was evaluated through 20 questions, each with four answer options and only one correct answer. The topics covered were related to the basis of genetics, such as genome (items 1, 3, 5, 10 and 13), cellular transcription, replication and translation (items 2, 8 and 14), chromosomal aberrations (item 12) and heritability (item 4 and 6). The clinical and therapeutic application of it were also included. For instance, genomics (item 7, 9, 11, 17, 19 and 20), gen technology (item 15 and 16) and diagnosis tests (item 18). The score was made on a scale of 0 to 100, where each correct answer was valued with 5 points. The results were classified into three categories: high (80 to 100 points), intermediate (55 to 75 points), and low (<50 points).

The last part was about population’s knowledge on the heritability of certain traits and diseases was also evaluated, including height, weight, intelligence, eye color, clinical depression, motivation, school performance, sexual orientation, attention-deficit/hyperactivity disorder (ADHD), dyslexia, and schizophrenia. Each of these topics was evaluated on a scale of 0 to 100, considering the underestimated and overestimated values of heritability according to the reference points established by Chapman et al. and other authors (21–30).

#### 2.4.3 Attitudes towards genetics testing

Attitudes towards genetic testing were evaluated through 10 questions. The first 9 questions about the topic were asked on 7-point Likert scales, which were subsequently grouped into three subgroups for analysis and interpretation. One scale was used to evaluate participants’ level of agreement, and another to evaluate the degree of probability. The last question of this section offered five response options about the main topic. The questions asked were focus on constitutional genetic testing (item 2, 8), clinical and therapeutic applications of genetics (item 9, 14), scientific advancements (item 7, 11), access to conventional medicine (item 6) and other general topics (item 3, 10, 13).

### 2.5 Statistical methods

The statistical analysis of the data was performed using IBM-SPSS v. 23 and Microsoft Excel 2016. Percentages and frequencies were used for categorical variables. For continuous variables with normal distribution according to the Kolmogorov–Smirnov test, means and standard deviations were employed. For continuous variables that did not have a normal distribution, the median and interquartile range were used.

### 2.6 Ethical approval and consent to participate

The Research Bioethics Committee of the Health Area of the University of Cuenca (COBIAS-UCuenca) approved the research protocol presented through the Resolution No. 144–09/3/2022. Moreover, the information used in this study was rendered anonymous.

## 3 Results

A cohort of 153 medical students underwent a survey to assess their knowledge and attitudes regarding genetics and genetic testing, respectively. The completion rate achieved was 100%, with all participants fully completing the questionnaire, and no responses were missing. Consequently, the primary findings of this study are outlined below.

### 3.1 Sociodemographic features

Approximately 58.2% (*n* = 89) of the participants were identified as female. The predominant age group ranged between 22 to 25 years old, constituting 85.0% (*n* = 130), with a median age of 24 years (IQR = 20 years) (Table 1).

**Table 1:** Sociodemographic characteristics of the final-year medical students.

Sociodemographic variables	*n*	%
Sex		
Female	89	58.17
Male	64	41.83
Age		
Median (IQR)^a^	24 (22–42)	
From 22 to 25 years old	130	84.97
From 26 to 30 years old	21	13.73
> 30 years old	2	1.31
Number of children		
0	142	92.81
1	6	3.92
2	5	3.27
Children <16 years old		
No	1	9.09
Yes	10	90.90
Religion		
No	8	8.50
Yes	145	91.50
Type of religion		
Agnostic	23	15.86
Atheist	5	3.45
Christian	60	41.38
No religion	13	8.97
Other religion	44	30.34

a Age did not have a normal distribution according to the Kolmogorov–Smirnov test performed.

In terms of previous exposure to genetics, respondents were allowed to select multiple options. A significant majority, 89.54% (*n* = 137) of students, reported having studied genetics as part of their university curriculum, while 4.58% (*n* = 7) indicated no interest in the subject. Conversely, individuals interested in genetics pursued self-study through various mediums such as documentaries, courses, and books, accounting for 8.5% (*n* = 13), while 7.84% (*n* = 12) followed genetic topics on social media.

### 3.2 Genetic knowledge self-assessment

The degree of genetic knowledge according to each participant had an average of 40.1 ± 17.57 SD on a scale of 0 to 100. 24.84% (*n* = 38) of students considered having an intermediate genetic knowledge with a score of 50 points. Meanwhile, 1.96% (*n* = 3) had zero or low knowledge on the subject. No participant considered their knowledge of genetics to exceed 90 or 100 points.

### 3.3 Genetic knowledge evaluation

Genetic knowledge was assessed using a 20-question evaluation with a total value of 100 points. The mean number of correct responses was 12.78 ± 2.23 SD, equivalent to a score of 63.9 ± 11.14 SD. The highest score obtained was 90 (1.31%; *n* = 2), with a total of 18 correct responses, while the lowest score was 35 (1.31%; *n* = 2), with only 7 correct responses.

Questions with the highest percentage of correct answers were related to the main function of genes (98.0%; *n* = 150), the base units that make up DNA (96.1%; *n* = 147), and the number of chromosomes in humans (95.4%; *n* = 146). In contrast, questions with the highest errors in response were those related to the heritability percentage of insomnia (88.24%; *n* = 135), the average total DNA similarity in two randomly selected individuals (79.74%; *n* = 98), and the DNA sequence in two different cells in the same person (69.93%; *n* = 107). Results indicated that 75.16% (*n* = 115) of participants had intermediate knowledge of genetics, while only 9.80% (*n* = 15) had a high level of knowledge (Table 2).

**Table 2:** Genetic knowledge: distribution of correct and incorrect answers.

Questions	Correct answers		Incorrect answers	
	*n*	%	*n*	%
Definition of genome	105	68.63	48	31.37
DNA base units	147	96.08	6	3.92
Gene copies in autosomal cells	63	41.18	90	58.82
DNA shared siblings	86	56.21	67	43.79
Genes function	150	98.04	3	1.96
DNA and randomly selected persons	31	20.26	122	79.74
Genetic contribution and schizophrenia	102	66.67	51	33.33
Number of chromosomes in humans	146	95.42	7	4.58
Definition of epigenetic changes	92	60.13	61	39.87
Number of genes in humans	53	34.64	100	65.36
Genetic contribution and autism	95	62.09	58	37.91
Definition of polymorphisms	140	91.50	13	8.50
Gene sequence in different cells	46	30.07	107	69.93
Function of non-coding DNA	114	74.51	39	25.49
Selective breeding and genetic engineering	117	76.47	36	23.53
Gene editing methods	54	35.29	99	64.71
Behavioural prediction and DNA	115	75.16	38	24.84
Genetic Testing and genetic traits	146	95.42	7	4.58
Dyslexia and ADHD genes	136	88.89	17	11.11
Heritability of insomnia	18	11.76	135	88.24

### 3.4 Knowledge of heritability of traits and diseases

**Figure 1:**
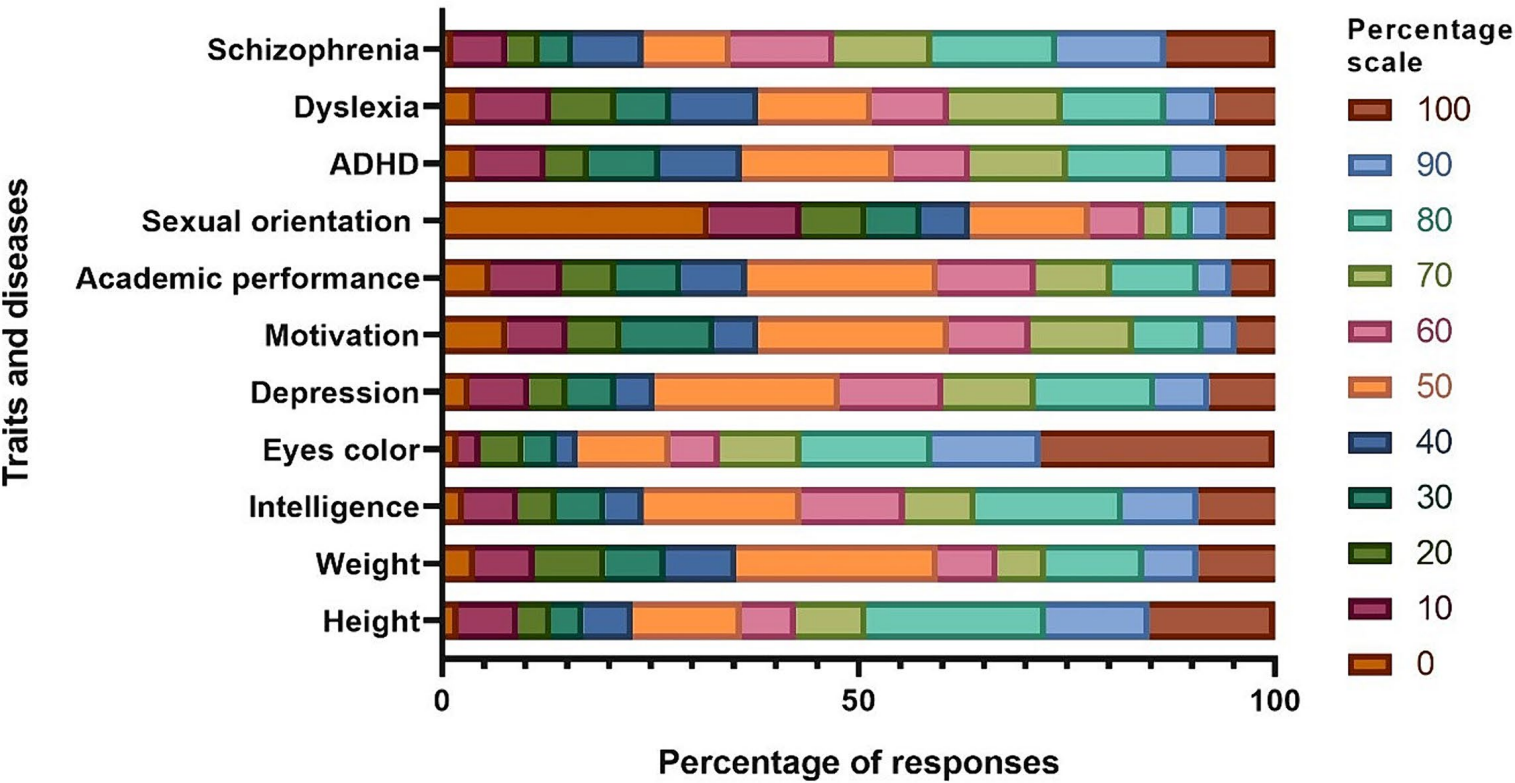
Estimated percentage of heritability for traits and diseases. ADHD, attention-deficit hyperactivity disorder.

Regarding the population’s knowledge of the heritability of certain traits and diseases, 32.03% (*n* = 49) believed that sexual orientation is not heritable (score of 0 points). On the other hand, 28.10% (*n* = 43) believed that eye color is heritable (score of 100 points). Participants underestimated the heritability percentages for weight (50%), academic performance (50%), ADHD (50%), and schizophrenia (70%). On the other hand, they overestimated the values for depression (60%) (Figure 1).

### 3.5 Attitudes towards genetic testing

A total of 49 respondents (32.03%) expressed distrust in the country’s research institutions due to the possibility of genetic data being misused, while 85 participants (55.56%) suspected that political or economic interests could influence genetic studies. On the other hand, 110 respondents (71.90%) agreed that genetic information should be used to adapt environmental conditions to individual needs, and 109 participants (71.24%) believed that gene editing should be permitted for the prevention and treatment of diseases. Moreover, 129 respondents (84.31%) agreed that scientific development is essential to improving people’s quality of life.

Regarding the use of gene editing to enhance or select specific traits in children, 49 respondents (32.03%) believed that parents should have the option (Table 3). In relation to the use of genetic engineering to treat rare disorders that are completely caused by genetic influences, 75 participants (49.02%) considered it appropriate, followed by pharmacological treatment (*n* = 40; 26.14%) and changes in lifestyle (*n* = 26; 16.99%) (Table 4).

**Table 3:** Attitudes and conformity related to 8 items of the questionnaire.

Items	Agreed		Neutral		Disagree	
	*n*	%	*n*	%	*n*	%
Research and misuse of data in Ecuador	48	31.37	56	36.60	49	32.03
Genetically modified foods and safety	37	24.18	41	26.80	75	49.02
Environmental conditions and genetic information	110	71.90	27	17.65	16	10.46
Genetic manipulation and diseases	109	71.24	26	16.99	18	11.76
Gene editing and traits	49	32.03	45	29.41	59	38.56
Genetic Studies and political/financial interests	85	55.56	41	26.80	27	17.65
Scientific development and quality of life	129	84.31	16	10.46	8	5.23
Genetic influence and agency	36	23.53	44	28.76	73	47.71

**Table 4:** Attitudes toward treatment of genetically influenced disorders.

Option	*n*	%
Lifestyle changes (e.g., diet)	26	16.99
Surgery	5	3.27
Pharmacological (medication)	40	26.14
Genetic engineering	75	49.02
Talking therapies (e.g., counselling)	7	4.58

Concerning genetic testing, 117 respondents (76.47%) agreed to undergo genetic testing if it allowed for better treatment of a specific condition. Moreover, 92 participants (60.13%) considered it unlikely to opt for alternative medicine methods such as homeopathy, naturopathy or chiropractic instead of conventional medicine when they get down with flu, headache, malaise and others health related problems. Additionally, 108 respondents (70.59%) would be willing to provide a DNA sample for research if their data were stored anonymously (Table 5).

**Table 5:** Attitudes and probabilities related to 3 items of the questionnaire.

Item	Likely		Neutral		Improbable	
	*n*	%	*n*	%	*n*	%
Genetic testing and treatment	117	76.47	12	7.84	24	15.69
Recourse to alternative medicine	42	27.45	19	12.42	92	60.13
DNA simple and anonymity	108	70.59	19	12.42	26	16.99

## 4 Discussion

Genetics has proven to be highly applicable in the field of medicine, serving as a valuable diagnostic, preventive, therapeutic, and surveillance tool for patients, their families, and the population. Knowledge of genetic diseases among healthcare professionals and early and targeted molecular diagnosis based on comprehensive genetic testing are essential in significantly reducing the economic burden and impact that genetic diseases represent for healthcare systems (31). Therefore, it is crucial that appropriate genetics education be provided during university training for future healthcare professionals, enabling them to develop accurate diagnostic criteria and properly interpret genetic test results. However, several studies have shown that healthcare professionals have poor understanding of genetics and genetic testing.

The present study evaluated the knowledge of genetics in a group of senior university students and found that they possessed an intermediate level of knowledge in the subject. It is important to note that only 15.04% of the participants had low knowledge about genetics, which is better than reported in previous studies. Alotaibi et al. (32) found that medical students at a Saudi university had insufficient knowledge of genetics, while Rujito et al. (33), Rojas-Betancourt et al. (34), and Lin et al. (35) observed inadequate levels of genetic knowledge, genetic diseases and genetic testing in undergraduate medical students at universities in Indonesia, Angola and Malaysia, respectively. Furthermore, Ramalle-Gõmara et al. (36) identified that only one-fourth of the future health care and non-health care professionals at a Spanish university were familiar with the definitions of rare diseases and orphan drugs. Additionally, Colotto et al. (37) and Fontenla et al. (11) discovered that less than half of Italian and Argentine medical students surveyed exhibited a lack of familiarity with genetic testing and the main risk factors for congenital malformations, correspondingly. Meanwhile, Hauser et al. (38) unveiled that three out of four primary care physicians in New York possessed inadequate knowledge regarding genetic testing and risk factors for chronic diseases. Similarly, Melo et al. (13) and Lópes-Júnior et al. (15) determined that approximately one-eighth of primary health care professionals in Brazil were acquainted with genetic and its terminology and fundamentals. However, three-quarters of the participants did not feel adequately equipped to provide genomic-based healthcare (15).

In contrast to prior investigations, the findings of this study showed a level of knowledge in genetics similar to that documented by Morales Ríos (10). About 67.0% of undergraduate medical students at the Autonomous University of Nuevo León (Mexico) demonstrated a medium knowledge of genetics, followed by high (28.0%) and low knowledge levels (5.0%) (10).

On the other hand, an underestimation of the heritability of certain traits and diseases such as academic performance, weight, ADHD, and schizophrenia was found, in contrast to the findings of Liu et al. (22), Rimfeld et al. (26), Chen et al. (28), and Merikangas et al. (30) who found different heritability percentages for academic performance ranging from 54 to 65%, weight from 63 to 87%, ADHD from 60 to 90%, and schizophrenia from 80 to 90%. However, estimates for other traits, such as height and dyslexia, were within the estimated percentage ranges of 70 to 90% and 40 to 80%, respectively, according to Doust et al. (29) and Liu et al. (22). It is important to note that most participants believed that sexual orientation is an inherited trait (27), whereas about 32.03% of the participants believed the opposite. It is now known that sexual orientation is the result of a complex interaction between genetic, hormonal and environmental factors (27, 39).

In our study, participants generally exhibited favorable attitudes towards genetic testing. However, a notable finding was the lack of confidence in the country’s research institutions among respondents, with nearly 30% refusing to provide a DNA sample for research purposes. Over recent years, genetic research in Ecuador has seen growth due to initiatives from the Ministry of Public Health, research projects in national universities, and activities undertaken by private institutions (40). Nevertheless, accessibility to these services remains limited, primarily targeting vulnerable populations at the public level, while private services face increasing costs and demand. It is imperative to emphasize that prior to undergoing genetic testing, patients must receive proper information and provide consent for the procedure under medical supervision. The Ecuadorian Ministry of Public Health, through the Integral Public Health Network and the Complementary Private Network, covers all expenses for diagnostic and research genetic tests, benefiting both insured and uninsured patients (41). The National Organic Health Law stipulates specific purposes for genetic testing, including establishing parentage, identifying genetic variants predisposing to diseases or affecting treatment response, and facilitating biomedical research projects (42).

Following the completion of these tests, the handling, reporting, retention, review, or disposal of results are dictated by the test’s purpose and the patient’s informed consent. Furthermore, biomedical research utilizing genetic data falls under the regulations of the National Organic Health Law (42). Unfortunately, while Ecuador has laws protecting personal data, there is presently no specific regulation governing the proper use, storage, and handling of biological samples for research purposes, often leaving the cost of these tests to be borne by the patients themselves.

Our findings resonate with previous studies by Eum et al. (43), Cheung et al. (44), and Abdul Rahim et al. (45), which similarly reported low levels of trust in research institutions among survey participants. Several factors contribute to this distrust and reluctance to contribute genetic material, including suspicions of political or economic interests influencing genetic studies, concerns regarding the privacy of genetic data, limited human resources and expertise in genetics, inadequate technological infrastructure for genetic and biomedical testing, and high costs associated with genetic tests. Despite these challenges, our findings suggest promising prospects for the study and research of human genetics in Ecuador (46).

Moreover, our study found similar results to those reported by Izzah et al. (47), Setyanto et al. (48), and Arias-Salazar et al. (49) regarding the use of gene editing and genetic engineering among medical students and healthcare professionals from Indonesia and Costa Rica. Specifically, less than half of the participants agreed with the use of gene editing to enhance or select specific traits in children, such as physical appearance, intelligence, strength, etc. (47, 48). On the contrary, more than three-quarters of the participants agreed with its use for the prevention and treatment of certain diseases (47–49). Additionally, about half of the participants opted for genetic engineering as a treatment for rare genetically caused disorders and other life-threatening and debilitating diseases (48, 49). Due to its rapid development and adoption as therapeutic options in managing common and rare diseases, a better and more extensive education on gene therapy and genetic engineering techniques is the explanation for these findings. Furthermore, Vockely et al. (50) consider this as a significant opportunity to enhance the training of genetic physicians and increase interest in medical genetics as a specialty.

Compared to previous studies, notable differences in knowledge of genetics were observed in this study. This may be due to participant selection, their level of advancement in the curriculum, and the structure and objectivity of the questionnaires used. However, it is evident that, regardless of general knowledge level of genetics, there is a lack of understanding about its clinical and therapeutic application. This is largely due to the way genetics is taught in HEIs. As previously mentioned, genetics constitutes part of the curriculum during the initial 3 years of medical education programs at Ecuadorian HEIs. It emphasizes the understanding of the fundamental principles and theoretical underpinnings of genetics. Considered as a basic science, genetics is taught alongside other courses such as Biology, Biochemistry and Immunology (18, 19). Subsequently, as medical students progress into their clinical and surgical training, genetics is not delved into extensively. Instead of this, the focus remains primarily on disease etiology in a broad manner. According to our literature review, this is not only a problem at Ecuadorian HEIs but also it extends beyond to an international scale. While curriculum design plays an important role, the lack of integration between genetics and clinical and surgical sciences represents a substantial challenge. Consequently, medical students perceive genetics as a science with very limited practical application and, therefore, it loses relevance.

### 4.1 Strengths and limitations of the study

In Latin America, unlike other regions of the world, there has been little research and publication on genetics teaching and learning. In Ecuador, no studies have evaluated knowledge and attitudes towards genetic testing among undergraduate medical students, graduated doctors, or healthcare professionals. Therefore, this study represents a significant contribution to the academic field at the regional, national, and international levels, and serves as a first approach to the topic among the targeted population.

One of the most significant limitations of the study is the lack of availability of the Rotating Internship Program students to respond to the survey. Additionally, the reliability of the responses obtained is compromised due to the length of the questionnaire and the time at which the participants responded (e.g., during work hours). Moreover, the fact that the students in the May 2021–April 2022 Cohort were only a few weeks away from completing their Rotating Internship generates a bias in their knowledge and attitudes towards genetics in clinical practice.

### 4.2 Further research

Suggestions for future research include exploring the relationship between the listed sociodemographic factors and knowledge and attitudes towards genetics and genetic testing. It is also recommended to include other important sociodemographic data such as socioeconomic status, province or region of origin and/or residence, the existence of genetic or non-communicable chronic diseases in the family tree, etc. Additionally, extending the study to undergraduate medical students would allow for an analysis of the distribution of knowledge and attitudes towards genetics and genetic testing in relation to the courses received, such as basic, clinical, and surgical courses. Finally, exploring knowledge and attitudes regarding ethical aspects of genetic testing and genetic therapies could be an interesting topic, because a vast debate has been taken part nowadays.

## 5 Conclusion

In conclusion, three-quarters of the medical students had intermediate knowledge about genetics, and their attitudes towards the clinical and therapeutic application of genetics, scientific development, access to conventional medicine, contribution to genetic studies, and support for genetic engineering for the treatment and management of genetically influenced disorders were appropriate. However, the current training of students in this field falls short of the demands of contemporary medicine. These findings suggest the need to evaluate the curriculum for genetics and propose pedagogical adjustments to improve students’ knowledge. Therefore, a comprehensive analysis and restructuring of the curricula in higher education institutions regarding the teaching of genetics and its relationship with clinical and surgical sciences is essential. This will help prepare students for the challenges brought about by medical technology in the 21st century and generate greater interest in the subject.

## Data availability statement

The raw data supporting the conclusions of this article will be made available by the authors, without undue reservation.

## Ethics statement

This study was conducted in accordance with the local legislation and institutional requirements. Due to the involvement of human participants, it received approval from the competent authorities within the institution, namely the Research Bioethics Committee of the Health Area of the University of Cuenca (COBIAS-UCuenca). Throughout its development, participants provided their electronic informed consent, which was included in the first section of the questionnaire. Finally, the information used was rendered anonymous.

## Author contributions

DJ-A: Conceptualization, Data curation, Formal analysis, Methodology, Project administration, Resources, Software, Validation, Visualization, Writing – original draft, Writing – review & editing. KS-R: Data curation, Formal analysis, Funding acquisition, Methodology, Software, Supervision, Validation, Visualization, Writing – original draft, Writing – review & editing.
