# Supplementary material for: Genetic knowledge and attitudes towards genetic testing among final-year medical students at a public university in Ecuador
Source: Front Med (Lausanne). 2024 Jun 19;11:1363552. doi: 10.3389/fmed.2024.1363552 (PMC11219587; doi:10.3389/fmed.2024.1363552)
Supplement: Supplementary file 1 [file Data_Sheet_1.PDF]

# *Supplementary Material*

## 1 Supplementary Figures and Tables

### 1.1 Tables

***Table 1.*** Sociodemographic characteristics of the final-year medical students.

| **Sociodemographic variables** | **n** | **%** |
|---|---|---|
| Sex | | |
| Female | 89 | 58.17 |
| Male | 64 | 41.83 |
| Age | | |
| Median (IQR)[a] | 24 (22 – 42) | |
| From 22 to 25 years old | 130 | 84.97 |
| From 26 to 30 years old | 21 | 13.73 |
| > 30 years old | 2 | 1.31 |
| Number of children | | |
| 0 | 142 | 92.81 |
| 1 | 6 | 3.92 |
| 2 | 5 | 3.27 |
| Children <16 years old | | |
| No | 1 | 9.09 |
| Yes | 10 | 90.90 |
| Religion | | |
| No | 8 | 8.50 |
| Yes | 145 | 91.50 |
| Type of religion | | |
| Agnostic | 23 | 15.86 |
| Atheist | 5 | 3.45 |
| Christian | 60 | 41.38 |
| No religion | 13 | 8.97 |
| Other religion | 44 | 30.34 |

[a] Age did not have a normal distribution according to the Kolmogorov-Smirnov test performed.

*Table 2.* Genetic knowledge: distribution of correct and incorrect answers.

| Questions | Correct answers | | Incorrect answers | |
|---|---|---|---|---|
| | n | % | n | % |
| Definition of genome | 105 | 68.63 | 48 | 31.37 |
| DNA base units | 147 | 96.08 | 6 | 3.92 |
| Gene copies in autosomal cells | 63 | 41.18 | 90 | 58.82 |
| DNA shared siblings | 86 | 56.21 | 67 | 43.79 |
| Genes function | 150 | 98.04 | 3 | 1.96 |
| DNA and randomly selected persons | 31 | 20.26 | 122 | 79.74 |
| Genetic contribution and schizophrenia | 102 | 66.67 | 51 | 33.33 |
| Number of chromosomes in humans | 146 | 95.42 | 7 | 4.58 |
| Definition of epigenetic changes | 92 | 60.13 | 61 | 39.87 |
| Number of genes in humans | 53 | 34.64 | 100 | 65.36 |
| Genetic contribution and autism | 95 | 62.09 | 58 | 37.91 |
| Definition of polymorphisms | 140 | 91.50 | 13 | 8.50 |
| Gene sequence in different cells | 46 | 30.07 | 107 | 69.93 |
| Function of non-coding DNA | 114 | 74.51 | 39 | 25.49 |
| Selective breeding and genetic engineering | 117 | 76.47 | 36 | 23.53 |
| Gene editing methods | 54 | 35.29 | 99 | 64.71 |
| Behavioural prediction and DNA | 115 | 75.16 | 38 | 24.84 |
| Genetic Testing and genetic traits | 146 | 95.42 | 7 | 4.58 |
| Dyslexia and ADHD genes | 136 | 88.89 | 17 | 11.11 |
| Heritability of insomnia | 18 | 11.76 | 135 | 88.24 |

***Table 3.*** Attitudes and conformity related to 10 items of the questionnaire.

| **Items** | **Agreed** | | **Neutral** | | **Disagree** | |
|---|---|---|---|---|---|---|
| | **n** | **%** | **n** | **%** | **n** | **%** |
| Research and misuse of data in Ecuador | 48 | 31.37 | 56 | 36.60 | 49 | 32.03 |
| Genetically modified foods and safety | 37 | 24.18 | 41 | 26.80 | 75 | 49.02 |
| Environmental conditions and genetic information | 110 | 71.90 | 27 | 17.65 | 16 | 10.46 |
| Genetic manipulation and diseases | 109 | 71.24 | 26 | 16.99 | 18 | 11.76 |
| Gene editing and traits | 49 | 32.03 | 45 | 29.41 | 59 | 38.56 |
| Genetic Studies and political/financial interests | 85 | 55.56 | 41 | 26.80 | 27 | 17.65 |
| Scientific development and quality of life | 129 | 84.31 | 16 | 10.46 | 8 | 5.23 |
| Genetic influence and agency | 36 | 23.53 | 44 | 28.76 | 73 | 47.71 |

***Table 4.*** Attitudes toward treatment of genetically influenced disorders.

| Option | n | % |
|---|---|---|
| Lifestyle changes (e.g., diet) | 26 | 16.99 |
| Surgery | 5 | 3.27 |
| Pharmacological (medication) | 40 | 26.14 |
| Genetic engineering | 75 | 49.02 |
| Talking therapies (e.g., counselling) | 7 | 4.58 |

*Table 5.* Attitudes and probabilities related to 3 items of the questionnaire.

| Item | Likely | | Neutral | | Improbable | |
|---|---|---|---|---|---|---|
| | n | % | n | % | n | % |
| Genetic testing and treatment | 117 | 76.47 | 12 | 7.84 | 24 | 15.69 |
| Recourse to alternative medicine | 42 | 27.45 | 19 | 12.42 | 92 | 60.13 |
| DNA simple and anonymity | 108 | 70.59 | 19 | 12.42 | 26 | 16.99 |

## 1.2 Figures

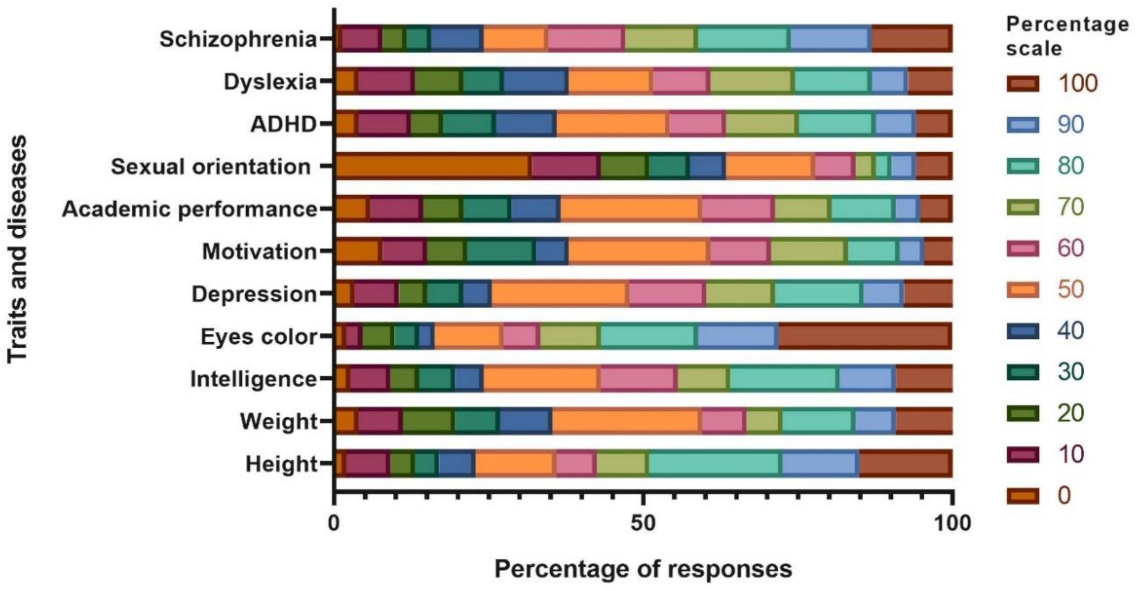


**Figure 1. Estimated percentage of heritability for traits and diseases**.
ADHD: Attention-Deficit Hyperactivity Disorder.
